# A basic phosphoproteomic-DIA workflow integrating precise quantification of phosphosites in systems biology

**DOI:** 10.52601/bpr.2023.230007

**Published:** 2023-04-30

**Authors:** Yi Di, Wenxue Li, Barbora Salovska, Qian Ba, Zhenyi Hu, Shisheng Wang, Yansheng Liu

**Affiliations:** 1 Cancer Biology Institute, Yale University School of Medicine, West Haven, CT 06516, USA; 2 Current address: Laboratory Center, Shanghai Municipal Hospital of Traditional Chinese Medicine, Shanghai University of Traditional Chinese Medicine, Shanghai 200071, China; 3 Department of Pulmonary and Critical Care Medicine, and Proteomics-Metabolomics Analysis Platform, West China Hospital, Sichuan University, Chengdu 610041, China; 4 Department of Pharmacology, Yale University School of Medicine, New Haven, CT 06510, USA

**Keywords:** Bioinformatic analysis, High-resolution mass spectrometry technology, Phosphoproteomics, Systems biology, Systems medicine

## Abstract

Phosphorylation is one of the most important post-translational modifications (PTMs) of proteins, governing critical protein functions. Most human proteins have been shown to undergo phosphorylation, and phosphoproteomic studies have been widely applied due to recent advancements in high-resolution mass spectrometry technology. Although the experimental workflow for phosphoproteomics has been well-established, it would be useful to optimize and summarize a detailed, feasible protocol that combines phosphoproteomics and data-independent acquisition (DIA), along with follow-up data analysis procedures due to the recent instrumental and bioinformatic advances in measuring and understanding tens of thousands of site-specific phosphorylation events in a single experiment. Here, we describe an optimized Phos-DIA protocol, from sample preparation to bioinformatic analysis, along with practical considerations and experimental configurations for each step. The protocol is designed to be robust and applicable for both small-scale phosphoproteomic analysis and large-scale quantification of hundreds of samples for studies in systems biology and systems medicine.

## INTRODUCTION

The functional diversity of the proteome largely depends on post-translational modifications (PTMs). In fact, the number of unique proteins or proteoforms is significantly larger than the number of genes in the human genome, largely because of PTMs (Aebersold *et al*. 2018; Jensen 2004). Moreover, protein activity is strongly influenced by PTMs, making it an urgent and challenging task to understand the impact of PTMs on protein function.

Protein phosphorylation is a reversible process that involves the modification of amino acid residues such as serine, threonine, and tyrosine, by the addition of phosphate groups derived from adenosine 5'-triphosphate (ATP) with the aid of kinases (Cohen 2002). Phosphorylation was also observed occurring at noncanonical amino acid residues such as histidine, which is remarkably widespread in bacteria (Potel *et al*. 2018). The prevalence of phosphorylation is high, with estimates suggesting that over two-thirds of proteins are phosphorylated, often at multiple sites. This leads to the occurrence of more than 100,000 distinct phosphorylation events identified in human cells (Needham *et al*. 2019), adding a staggering amount of complexity to the proteome. It has been reported that phosphorylation modulates the function of proteins by altering various protein properties, including activity (Humphrey *et al*. 2015), structure, thermal stability (Huang *et al*. 2019), localization (Krahmer *et al*. 2018) and interaction (Betts *et al*. 2017). Also, dysregulated phosphorylation has been proven a hallmark in cancers (Blume-Jensen and Hunter 2001; Rikova *et al*. 2007; Zanivan *et al*. 2013) and Alzheimer’s disease (Eidenmüller *et al*. 2001; Grundke-Iqbal *et al*. 1986), underscoring its crucial role in cellular physiology and disease.

Over the past decade, mass spectrometry (MS) has emerged as the premier tool for analyzing proteins and their PTMs (Aebersold and Mann 2016) due to its high sensitivity, reproducibility, considerable throughput, and the capacity to identify high-confidence PTM sites. In a typical phosphoproteomics workflow, proteins are first extracted from biological samples and subjected to enzymatic digestion by, *e*.*g*., trypsin. Phosphorylated peptides are then normally enriched from the peptide mixture using metal ions such as titanium dioxide (TiO2) or chemically modified beads/columns (Low *et al*. 2021). So far, most of the phosphoproteomic studies have been conducted using the traditional data-dependent acquisition (DDA) “shotgun” approach. In DDA mode, the mass spectrometer first performs a survey scan for all the ionized precursors, and then selects the most-abundant precursors for MS2 sequencing. Thus, this real-time selection of precursors may cause the under-sampling issue, restrict the detection of dynamic range, and lack the analytical resolution in discriminating phosphopeptide isomers (*i*.*e*., the same peptide sequence with different phosphosite localizations), particularly in studies involving large sample cohorts. Considering the limitations of DDA above, data-independent acquisition (DIA) (Gillet *et al*. 2012; Venable *et al*. 2004) has emerged as a powerful alternative method for proteomics and PTMs analysis. Unlike DDA, in DIA all the coexisting precursors in the prespecified *m*/*z* ranges (so called windows), rather than those high-abundant ones, are isolated and fragmented together. This feature essentially allows DIA to “detect” all the “detectable” peptides including their common and unique PTM site-containing fragmental ions with the additional information of the liquid chromatographic co-elution pattern between all the precursors and fragmental ions deriving from the same peptidoform (Liu 2022; Rosenberger *et al*. 2017). Recent reports benchmarking DIA against DDA demonstrated greater quantitative accuracy and consistency of DIA in phosphoproteomics studies (Bekker-Jensen *et al*. 2020; Hoffman *et al*. 2015; Lawrence *et al*. 2016; Peckner *et al*. 2018; Rosenberger *et al*. 2017; Wang *et al*. 2020).

Our lab has also applied Phos-DIA in systems biological and systems medical studies. In the applications for systems biology, we first accomplished a benchmarking work, in which we profiled six distinct melanoma cell lines at proteome and phosphoproteome levels; and ~8,100 proteins and ~40,000 phosphopeptides were quantified using DIA-MS. We interrogated the absolute and relative independence of phosphopeptide abundances on protein levels (Gao *et al*. 2021). Moreover, we have described DeltaSILAC (delta determination of turnover rate for modified proteins by SILAC), a pilot effort systematically illustrating the impact of PTM sites on protein lifetime (Wu *et al*. 2021). DeltaSILAC is based on a combination of DIA-MS and the pulse experiment of stable isotope-labeled amino acids in cells (pulse SILAC) (Jovanovic *et al*. 2015; Liu *et al*. 2016; Schwanhausser *et al*. 2011). In DeltaSILAC, a paired strategy between the phosphorylated form and the unmodified form for the same peptide sequence was applied, resulting in a delta lifetime value (Δ*T*_1/2_) for site-specific phosphorylation. We have recently extended the lifetime-centric calculation in the steady state into the dynamic process, and proposed to convert the curving fitting problem to a time-series data analysis problem when analyzing the turnover difference between PTM-carrying peptides and corresponding non-PTM peptidoforms (Li *et al*. 2022). Besides phosphoproteome turnover analysis, we have performed an evolutionary profiling of phosphoproteomics in 11 common mammalian species (Ba *et al*. 2022). We found that compared to mRNA and protein layer, phosphosites are molecularly more dynamic. And for 611 common phosphosites across 11 species, quantitative results also highlighted the conservative and essential functions in the evolution process, with motifs significantly enriched, such as (SP), (SP.R) and (R.S). A phosphorylation coevolution network independent of protein abundance was also built in this study.

In the applications for systems medicine, to investigate the effect of metformin on cell signaling in colorectal cancer (CRC), we conducted an integrated analysis of phosphoproteomics, bioinformatics, and cell proliferation assays using a panel of 12 molecularly heterogeneous CRC cell lines (Salovska *et al*. 2023). In total, 10,142 proteins and 56,080 phosphosites were detected, revealing that while metformin only has a subtle regulatory effect on total protein expression levels, the response of the phosphorylation signaling pathway is much stronger and strikingly variable. We also examined the therapeutic potential of the metformin-altered phosphorylation network and confirmed a number of candidate metformin-interacting drugs, including navitoclax, a BCL-2/BCL-xL inhibitor.

In addition to the biological investigations above, we focused on bioinformatics for Phos-DIA. We co-developed a free and interface-friendly software called motifeR for conveniently identifying peptide/protein PTM motifs (Wang *et al*. 2019). Notably, phosphoproteomic datasets, even measured by DIA-MS, often have a higher prevalence of missing values compared to bulk-protein quantification due to the additional analytical challenges involved. For instance, phosphorylated sites are often characterized by low abundance and wide dynamic range. More importantly, high-confidence detection of fragment ions carrying phosphate is the prerequisite for phosphorylation location, which is much more challenging for large-scale analysis across many samples. We led the development of NAguideR (Wang *et al*. 2020), a standalone toolkit with a user-friendly interface and robust capabilities. NAguideR allows for the assessment and application of a variety of missing value imputation algorithms and guides the users to select one of the 23 common imputation methods using empirical proteomic evaluation criteria.

## PROTOCOL OVERVIEW

Building on the analytical superiority of DIA for phosphoproteomics analysis, we present here an optimized and feasible pipeline for DIA-based phosphoproteomics. Our present protocol represents biological, technical, and informatic considerations for applying Phos-DIA. The transient nature of phosphorylation and an instant response of the cells to certain stimuli require a rapid quenching of the investigated cellular phosphoproteome. We achieve this by snap-freezing the cell lines directly on the cell culture dishes, which enables harvesting cells after very short time intervals such as 15 or 30 s following a stimulation. Moreover, due to the dynamic nature of the phosphoproteome and the relatively low stoichiometry of this modification, the phosphoproteome analysis requires an efficient and cost-effective enrichment of phosphorylated peptides. In our workflow, the C18-purified peptides resulting from, *e*.*g*., precipitation-based tryptic digestion are subjected to a phospho-enrichment step using the high-select Fe-NTA kit. To this end, we first optimized the peptide-to-bead ratio and split the beads contained in one spin column from the kit into five aliquots, each one of them used to enrich ~250 µg of starting tryptic peptide material. In addition, we use filter-tips or filter plates to remove the flow-through after binding the phosphopeptides to the beads, to wash the beads, and elute the phosphopeptides using centrifugation or a vacuum manifold, respectively. These steps reduce the costs of the sample preparation and allow for the parallel processing of up to 96 samples together in one batch.

As described above, compared to DDA, the emerging data-independent acquisition (DIA) (Gillet *et al*. 2012; Venable *et al*. 2004) offers higher reproducibility and improved data completeness (Aebersold and Mann 2016; Collins *et al*. 2017; Liu *et al*. 2015, 2016). Due to the advances in software development, DIA-MS can be now used for PTM analysis, including protein phosphorylation (Bekker-Jensen *et al*. 2020; Demichev *et al*. 2020; Rosenberger *et al*. 2017). Furthermore, the focus on peptide-fragment ion level (MS2) information in phosphoproteomics-DIA supports a confident PTM-site identification, localization, and quantification (Bekker-Jensen *et al*. 2020; Rosenberger *et al*. 2017; Wang *et al*. 2020). Our optimized phosphoproteomic workflow (Ba *et al*. 2022; Gao *et al*. 2021; Salovska *et al*. 2023; Wu *et al*. 2021) takes the above advantages of DIA-MS. Moreover, for certain phosphorylation samples allowing multiple injections, we apply our BoxCarmax DIA-MS method (Salovska *et al*. 2021) which utilizes small DIA *m*/*z* windows and both MS1- and MS2-level multiplexing to dramatically improve the analytical selectivity for highly complex samples. This strategy is especially beneficial for the localization and quantification of different peptidoforms (Liu 2022) by separating the peptide ions into different DIA windows. Importantly, in experiments using SILAC or pulsed SILAC quantification such as the analysis of protein and phosphopeptide turnover (Salovska *et al*. 2020; Wu *et al*. 2021), the separation of the different channels into smaller BoxCarmax windows dramatically improves the quantification accuracy (Salovska *et al*. 2021).

During Phos-DIA data analysis, the PTM Workflow in Spectronaut (Bekker-Jensen *et al*. 2020) is used for raw data processing in this protocol. We present an example in which we use two different PTM Probability Cutoffs to export the phospho-precursor-level data (Gao *et al*. 2021). Also, we perform the selection of the most representative phospho-precursor for each P-site based on abundance and data completeness. We recognize and emphasize the importance of a P-site-specific analysis rather than the gene-specific analysis to derive biological insights from the phosphoproteomic data and provide a protocol for how to perform a P-site-specific enrichment analysis in Perseus (Cox and Mann 2012; Tyanova and Cox 2018; Tyanova *et al*. 2016). Furthermore, we devised several software tools for the downstream analysis of proteomic and phosphoproteomic data. First, the NAguideR can be used for data normalization, filtering, and missing values imputation (Wang *et al*. 2020). Second, StatsPro can be used to perform the differential expression analysis (Yang *et al*. 2022). Third, motifeR can be used to extract sequence motifs surrounding P-sites of interest, perform motif enrichment analysis, visualize the results, and visualize kinase signaling networks (Wang *et al*. 2019). These software tools are provided both in the form of R packages and user-friendly web-based applications by us and others.

**Figure 1:**
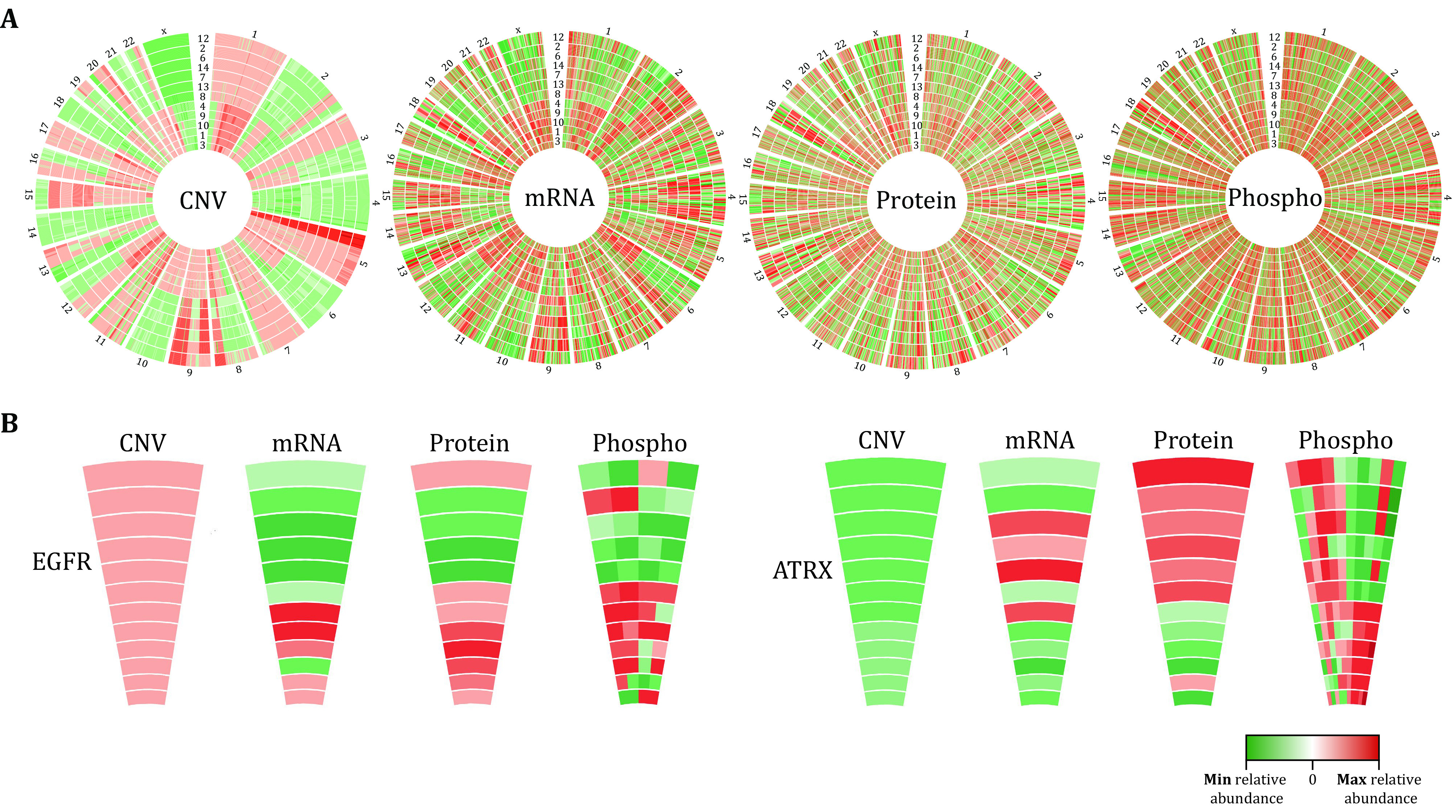
The heterogeneity of steady-state phosphorylation profiles across 12 HeLa cell lines emphasizes the importance of p-site-specific analysis to understand cellular functions. **A** Circos plot shows the quantitative differences in CNV, mRNA, protein, and phosphorylation levels across the 12 HeLa cells after normalizing the data to the mean of the respective values. **B** EGFR and ATRX are shown as representative examples

Finally, it should be stressed that, most phosphoproteomic analyses like our Phos-DIA workflow described here all assume that individual phosphorylation sites, rather than phosphorylated proteins, carry distinctive functions (Fig. 1, below). Therefore, site-specific phosphorylated peptidoforms (Liu 2022) are separately measured by MS and then individually annotated and analyzed by following up software algorithms such as motifeR and NAguideR. Take our previously published 14 Hela strains across different laboratories (Liu *et al*. 2019) as an example, the phosphoproteomic measurement of these cells (Wu *et al*. 2021) further supported their heterogeneity in gene expression and cell signaling (Fig. 1A). Particularly, compared to the gene-specific measurement of DNA copies, mRNA, and protein abundances, the phosphorylation measurement is site-specific (Fig. 1B), revealing a complexity that cannot be collapsed to the genes/protein level.

## MATERIALS AND EQUIPMENT

### Reagents

• Ammonium Bicarbonate: Thermo Fisher Scientific, Cat. #393212500

• Acetonitrile: Thermo Fisher Scientific, Cat. #51101

• Ammonium hydroxide solution: Sigma, Cat. #17093

• Acetic acid: Fisher Scientific, Cat. #A35-500

• Acetone: Fisher Scientific, Cat. #60-002-09

• Ethanol: Fisher Scientific, Cat. #AC611050040

• BioRad Bradford protein assay kit: BioRad, Cat. #5000201

• Formic acid: Fisher Scientific, Cat. #A117-50

• HPLC water: Fisher Chemical, Cat. #W64

• Urea: Sigma-Aldrich, Cat. #U5128

• PBS: Thermo Fisher Scientific, Cat. #10010023

• Protease inhibitor: Roche, Cat. #4693116001

• Phosphatase inhibitor: Thermo Fisher Scientific, Cat. #78428

• High-Select Fe-NTA kit: Thermo Fisher Scientific, Cat. #A32992

• C18 column: MarocoSpin Columns, NEST Group INC

• Methanol: Honeywell, Cat. #LC230-1

• Tip with filter: Axygen, part #TF-20-L-R-S

• Trifluoroacetic acid: Fisher Scientific, Cat. #A116-50

• DTT: DL-Dithiothreitol: Sigma, Cat. #D0632

• IAA: Iodoacetamide: Sigma, Cat. #I1149

• Trypsin: Promega Sequencing Grade Modified Trypsin, Cat. #V5111

• Scraper: Biologix, Cat. #702180

• 96-well plate for digestion: The Nest Group, Cat. #AS-968820, 2 mL maximum capacity

• C18 96-well plate: The Nest Group, Cat. #SNS-SS18VL, 350 μg maximum capacity

• 96-well plate with filter: PALL, Cat. #8231

### Buffer

• AmBic buffer: 100 mmol/L ammonia bicarbonate in HPLC water

• Cell lysis buffer: 10 mol/L urea in 100 mmol/L AmBic buffer with protease (one tablet in 40 mL) and phosphatase inhibitors (100× )

• Precipitation buffer: 50% ethanol + 49.9% acetone + 0.1% acetic acid

• Buffer A: 2% acetonitrile + 97.9% HPLC water + 0.01% formic acid

• Buffer B: 80% acetonitrile + 19.9% HPLC water + 0.01% formic acid

• Binding/Washing buffer: 80% acetonitrile + 19.9% HPLC water + 0.01% trifluoroacetic acid

• Elution buffer: 50% acetonitrile + 45% HPLC water + 5% ammonia solution

### Equipment

• Orbitrap Fusion Lumos Tribrid mass spectrometer, Thermo Scientific

• EASY-nanoLC 1200 systems, Thermo Scientific

• VialTweeter device, Hielscher-Ultrasound Technology

• Nanodrop, Thermo Scientific

• SPD111V, Thermo Scientific

• VORTEMP 56, Labnet

• SUPELCO vacuum manifold system

• ThermoMixer, F2.0

### Software

• Spectronaut v16, Biognosys, Inc. (Bruderer *et al*. 2015; Bruderer *et al*. 2017)

• R (version 3.6.0), R Core Team

• Perseus (version 1.6.14.0) (Tyanova and Cox 2018; Tyanova *et al*. 2016)

• NAguideR (v1) (Wang *et al*. 2020)

• StatsPro (v1) (Yang *et al*. 2022)

• motifeR (v1) (Wang *et al*. 2019)

### Data availability

The data used to generate Figs. 1 and 5 were extracted from our previously published dataset (Wu *et al*. 2021), for which all the raw data of mass spectrometry measurements can be freely downloaded from ProteomeXchange Consortium via identifier PXD017496.

## STEP BY STEP PROCEDURE

### Step 1: Cell harvest by snap-freezing on cell culture dishes [TIMING ~10 min]

**Figure 2:**
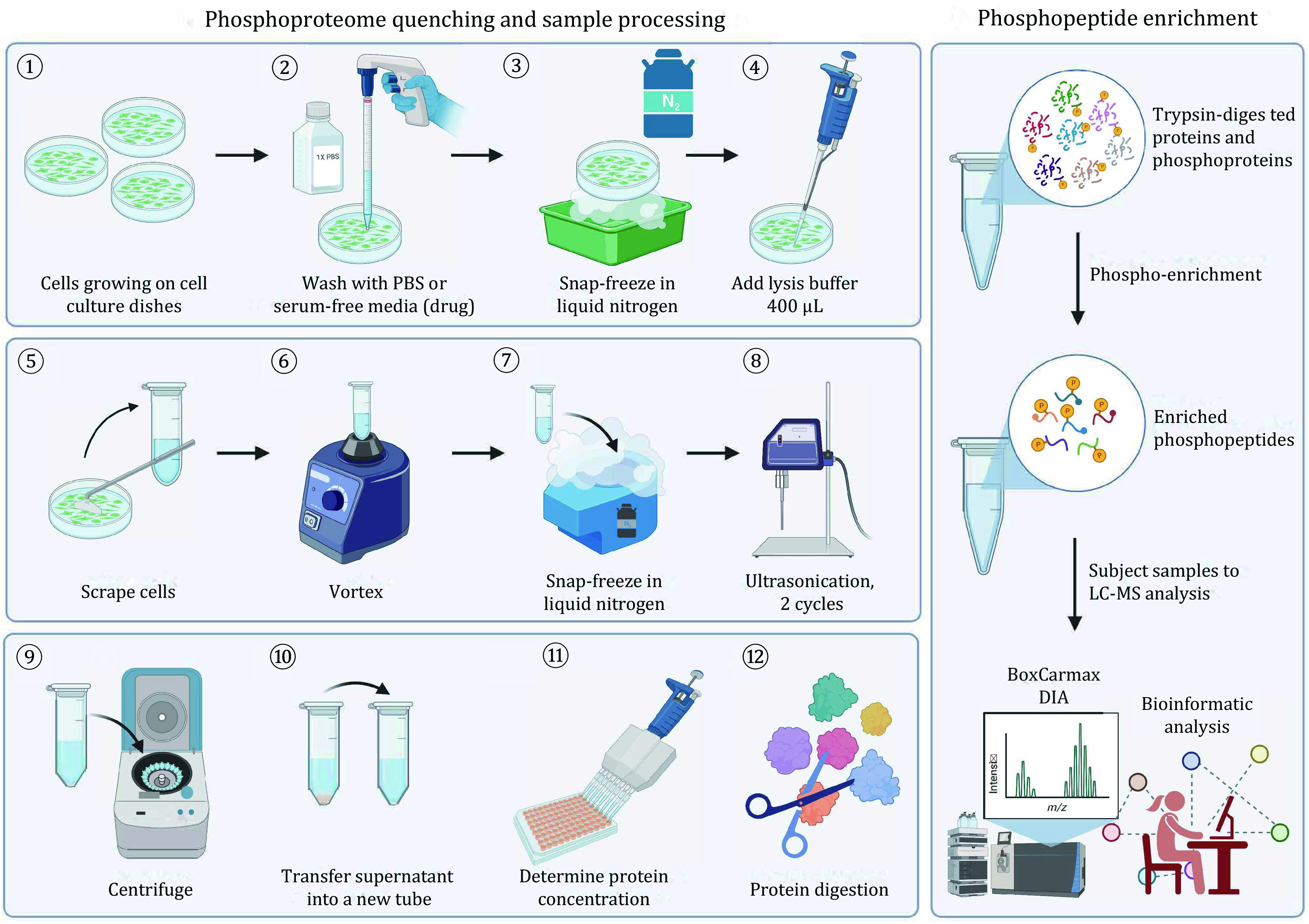
The overview of an optimized protocol for a rapid phosphoproteome quenching, phosphopeptide enrichment, and Phos-DIA-MS analysis using the BoxCarmax method. **A** Rapid phosphoproteome quenching. **B** Phosphoproteome enrichment

**[TIP]** Herein we describe an example in which we apply a rapid method to quench the phosphoproteome of cells growing on cell culture dishes by snap-freezing the whole dishes in liquid nitrogen. The rapid phosphoproteome quenching is extremely important for studies investigating the rapid phosphoproteome response to treatments occurring in seconds to minutes after stimulation. Please see Fig. 2 for an example of the experimental schema.

1.1 Start with a cell culture dish at *e*.*g*., 80% confluence and gently rinse the cells twice with 10 mL of precooled PBS to fully remove the serum.

1.2 After removing all the liquid, put the bottom of the dishes on the liquid nitrogen for 1 min.

**[TIP]** Some cell culture dishes are not suitable for this step and would break at low temperature. Thus, please test the dishes with liquid nitrogen before the harvest.

1.3 Add 400 μL lysis buffer to each dish and scrape the cells into a 2-mL Eppendorf tube.

1.4 Freeze the sample tubes in liquid nitrogen again.

**[TIP]** The experiment can be paused here. Transfer and store the samples at –80 °C for short storage.

### Step 2: Protein extraction [TIMING ~3 h]

2.1 Thaw the samples on ice. Perform the cell lysis by sonication, *e*.*g*., the Hielsher Vialtweeter instrument for 2 cycles, each cycle 1 min.

2.2 Centrifuge the cell lysates at 20,000 *g ×* 1 h, at 4–7 °C. Note, if the urea crystallization was observed, the centrifugation step can be performed at 20 °C.

2.3 Carefully transfer the supernatant to new 2-mL Eppendorf tubes. Discard the pellets containing insoluble material.

2.4 Perform protein concentration assay by, *e*.*g*., BCA protein assay kit, as described by the manufacturer. Then transfer 700 μg protein of each sample to a new Eppendorf tube for digestion.

**[TIP]** A minimum of 300 μg protein is needed per sample in the present workflow.

2.5 Reduction. Add 10 mmol/L DTT into each sample and incubate for 1 h at 56 °C.

2.6 Alkylation. Add 20 mmol/L IAA into each sample and keep in dark for 45 min at room temperature.

### Step 3: Protein precipitation (optional) [TIMING ~1 d]

**[TIP]** This step is performed for samples including reagents or buffers incompatible to MS analysis, such as SDS.

3.1 After reduction and alkylation, add five times volume of precipitation buffer to the protein mixture; keep at –20 °C for overnight.

3.2 Centrifuge 20,000 *g ×* 1 h, at 4 °C to collect the protein precipitate; discard the supernatant.

3.3 Wash the pellets first by precooled 100% acetone and then 70% ethanol, centrifuge 20,000 *g* × 40 min, at 4 °C.

3.4 Dry the pellet in SpeedVac for 5 min to remove the remaining organic solvents.

### Step 4: Protein digestion and peptide purification [TIMING ~1 d]

4.1 Add 300 μL 100 mmol/L AmBic buffer to the dried protein pellet.

**[TIP]** If the in-solution digestion is chosen (rather than precipitation), dilute the urea concentration to less than 2 mol/L.

4.2 Add 1:25 (*w*/*w*) trypsin for digestion at 37 °C overnight, shake at 1000 r/min.

**[TIP]** The trypsin is stored in an acidic buffer, after adding the trypsin into the sample, check the pH, and make sure the pH is below 7.

4.3 Equilibrate the C18 column sequentially by 300 μL methanol two times, Buffer B three times, Buffer A four times. Centrifuge at 300 *g* for 1 min.

4.4 Load the samples into the equilibrated column and reload three times.

4.5 Wash the column with 300 μL of Buffer A four times.

4.6 Elute peptides with 120, 100, and 80 μL of Buffer B sequentially, then combine the eluates, and dry in SpeedVac.

4.7 Measure the peptide concentration with NanoDrop, and then take an aliquot corresponding to 250 μg of peptides and dry again with SpeedVac.

### Step 4*: A large-scale version of protein digestion and peptide purification [TIMING ~1 d]

**[TIP]** For 100–1000 samples, we recommend that instead of above Step 4, the large-scale sample processing can be performed using 96-well plates (1. 96-well plate for digestion: The Nest Group, Cat. #AS-968820, 2 mL maximum capacity; 2. 96-well plate for desalting purification: The Nest Group, Cat. #SNS-SS18VL, 350 μg maximum capacity), including sample precipitation (optional), protein digestion, and peptide desalting purification. If the precipitation step is conducted in 2-mL tubes, our recommendation is to suspend the protein pellets in 100 μL 6 mol/L urea buffer, and then transfer the protein from the tube to a 96-well plate for downstream dilution, digestion, and purification. Please see the supplementary Fig. S1 for the experimental configuration using 96-well plates.

4.1* For the in-solution digestion: perform the reduction (10 mmol/L DTT) and alkylation (20 mmol/L IAA) in the plate as described in Steps 2.4–2.6. After that, dilute the sample with 100 mmol/L AmBic buffer until the urea concentration is less than 2 mol/L. For the precipitated protein mixture, dilute the sample with 300 μL 100 mmol/L AmBic buffer until the urea concentration is less than 2 mol/L.

4.2* Add 1:20 (*w*/*w*) trypsin for digestion at 37 °C (see the supplementary Fig. S1, Labnet instrument for large-scale digestion) overnight. Shake the plate at 1000 r/min.

**[TIP]** Trypsin is stored in the acid buffer. Therefore, after adding the trypsin into the sample, check and make sure that the pH value is >7.

4.3* C18 96-well plate coupled to the vacuum manifold (SUPELCO) system is used for the peptide desalting purification. Assemble the large-scale desalting system as shown in the supplementary Fig. S1. Slowly decrease the pressure by the valve and ensure the pressure is not lower than –40 kPa.

**[TIP]** Vent slowly also to reduce the liquid splash in the collection plate. To equilibrate of C18 96-well plate, put a 2-mL 96-well collection plate into the vacuum manifold and then a C18 plate at the top of the instrument. Use a 12-channel pipette to transfer the buffer into a plate, and sequentially wash the plate with methanol two times, Buffer B three times, and Buffer A four times. For each washing step, the recommended buffer volume is 200 μL.

4.4* Replace a new 2-mL 96-well collection plate in the vacuum manifold. Load the sample into an equilibrated C18 96-well plate.

**[TIP]** For the sample with a volume less than 200 μL, reloading one time for a total of two times is suggested.

4.5* Replace with another 2-mL 96-well collection plate and wash off the non-specific peptides by cleaning the C18 plate with 200 μL Buffer A for a total of four times. For highly salty samples, increasing wash times is needed.

4.6* Peptide elution. Replace a new 2-mL 96-well collection plate, and then peptides are eluted by Buffer B with 200, 150 and 100 μL, separately. Combine the eluates.

4.7* Peptide elution is dried by SpeedVac, ~3 h.

### Step 5: Phosphopeptide enrichment [TIMING ~1 d]

**[TIP]** 150–250 μg of peptides is an optimal starting amount for phosphopeptide enrichment. Reducing the input peptide amounts further tends to lead to a significant decrease in identified phosphopeptides in our hands.

5.1 Take 150–250 μg of purified peptide and resuspend it in 100 μL binding/wash buffer. Sonicate samples when necessary.

5.2 Beads equilibration. High-Select Fe-NTA kit is used for the phosphopeptide enrichment. Centrifuge the original spin column from the kit at 1000 *g* for 30 s to remove the storage buffer. Wash the spin column with binding/washing buffer three times, each time with 200 μL. Add 500 μL binding/washing buffer to one spin column, mix well and divide it into five equal aliquots; each 100 μL beads of suspension is sufficient for one sample enrichment.

**[TIP]** The recommended beads amount for 150–250 μg of total peptides is 1/5 volume of the original spin column. However, the enrichment recovery may vary from sample to sample. Just as an example, we normally can enrich ~5 μg phosphopeptides from 200 μg cancer cell-derived peptides using the settings here.

5.3 Add 100 μL beads slush into each sample and mix it well, incubate for 30 min at room temperature, and gently shake it per 10 min. Meanwhile, assemble the filter tip (Axygen, Cat. #TF-20-L-R-S) with a holder (holder is from Nest Group C18 column, Cat. #SUM-SS18V) and then put it on top of 2-mL tubes.

5.4 After incubation, the peptide binding beads are transferred into a filter tip. The buffer is spined down by centrifugation at 500 *g* for 30 s.

5.5 Wash the filter tips three times with 200 μL of binding/washing buffer, then twice with 200 μL of HPLC water, sequentially, to reduce the binding of non-phosphorylated peptides.

**[TIP]** Process as fast as possible so that beads drying could be avoided in all treatment steps.

5.6 Eluted the phosphopeptides twice with 100 μL of elution buffer, combine the eluates, and dry in SpeedVac immediately.

5.7 Measure phosphopeptide concentration by NanoDrop; use 1–2 μg of phosphopeptides for LC-MS analysis.

### Step 5*: A large-scale version of phosphopeptide enrichment [TIMING ~1 d]

**[TIP]** For the large-scale phosphopeptide enrichment, the 96-well plate with 1 μm filter (PALL, Cat. #8231) (called filter plate) is used to replace the single filter tip in order to increase the throughput. The vacuum manifold system (SUPELCO) is also used to wash and elute the phosphopeptides in this step.

5.1* Resuspend the purified peptide in the plate with 100 μL binding/wash buffer for each well. Sonication is necessary by putting the plate in the water bath sonicator for 5 min.

**[TIP]** For each well of the 96-well plate, 150–250 μg dried peptides is required for the following enrichment.

5.2* Beads equilibration. High-Select Fe-NTA kit is used for the phosphopeptide enrichment. Centrifuge the original spin column from the kit at 1000 *g* for 30 s to remove the storage buffer. Wash the spin column with 200 μL binding/washing buffer three times. Add 500 μL binding/washing buffer to one spin column, mix well and divide it into five equal aliquots, each 100 μL bead of suspension is sufficient for one sample enrichment. Totally, one 96-well plate sample needs ~20 Fe-NTA kits.

5.3* Add 100 μL beads slush into each well and mix it well, incubate for 30 min at room temperature, and gently shake it per 10 min.

5.4* After incubation, transfer the peptide-binding beads into a filter plate and put the plate on the top of vacuum manifold system. Then put a new 2-mL 96-well collection plate into the vacuum manifold. Slowly reduce the pressure with the vacuum valve till all liquid goes through the plate (see the supplementary Fig. S1).

**[TIP]** The vent step needs to be conducted slowly in case the liquid/beads splash. The flow through, which contains non-phosphorylated peptides, can be collected for further experiments if needed.

5.5* Replace a new collection plate, wash the filter plate three times with 500 μL of binding/washing buffer and two times with 500 μL of HPLC water sequentially, to reduce the binding of non-phosphorylated peptides.

5.6* Replace another new collection plate, eluted the phosphopeptides twice with 100 μL of elution buffer, combine the eluates, and dry in SpeedVac immediately (Phosphorylation peptide sample is unstable to be stored a long time at high pH elution buffer (pH > 10)).

5.7* Measure phosphopeptide concentration by NanoDrop; use 1–2 μg of phosphopeptides for LC-MS analysis.

### Step 6: DIA-MS analysis — BoxCarmax-DIA-MS data acquisition [TIMING ~6 h]

**[TIP]** The MS setting reported below is specific to the Orbitrap Fusion Lumos. The parameters might need to be adjusted for other instruments. The BoxCarmax method consists of four injections, called here 1^st^, 2^nd^, 3^rd^, and 4^th^. Please refer BoxCarmax method on paper (Salovska *et al*. 2021). The readers can also download the raw data from PRIDE PXD021922 (https://proteomecentral.proteomexchange.org/cgi/GetDataset?ID=PXD021922) to import the method in Xcalibur. Both MS1 and MS2 spectra were recorded in a profile mode.

6.1 NanoLC setting. 100% water with 0.1% formic acid (A); 80% acetonitrile + 20% water with 0.1% formic acid (B). The running time of one injection is 60 min with a 300-nL/min flow rate. The 50 cm length column (75 μm I.D.) with a 5-μm emitter is used. The gradient starts from 6% B to 37% B in the beginning period of 37 min and then increases to 100% B in another 3 min. The last 8 min is kept at 100% B for washing column.

6.2 Global parameters. The positive voltage is 2000 V and the ion transfer tube temperature is 275 °C. The advanced peak determination is on, and the default charge state is 2.

**Figure 3:**
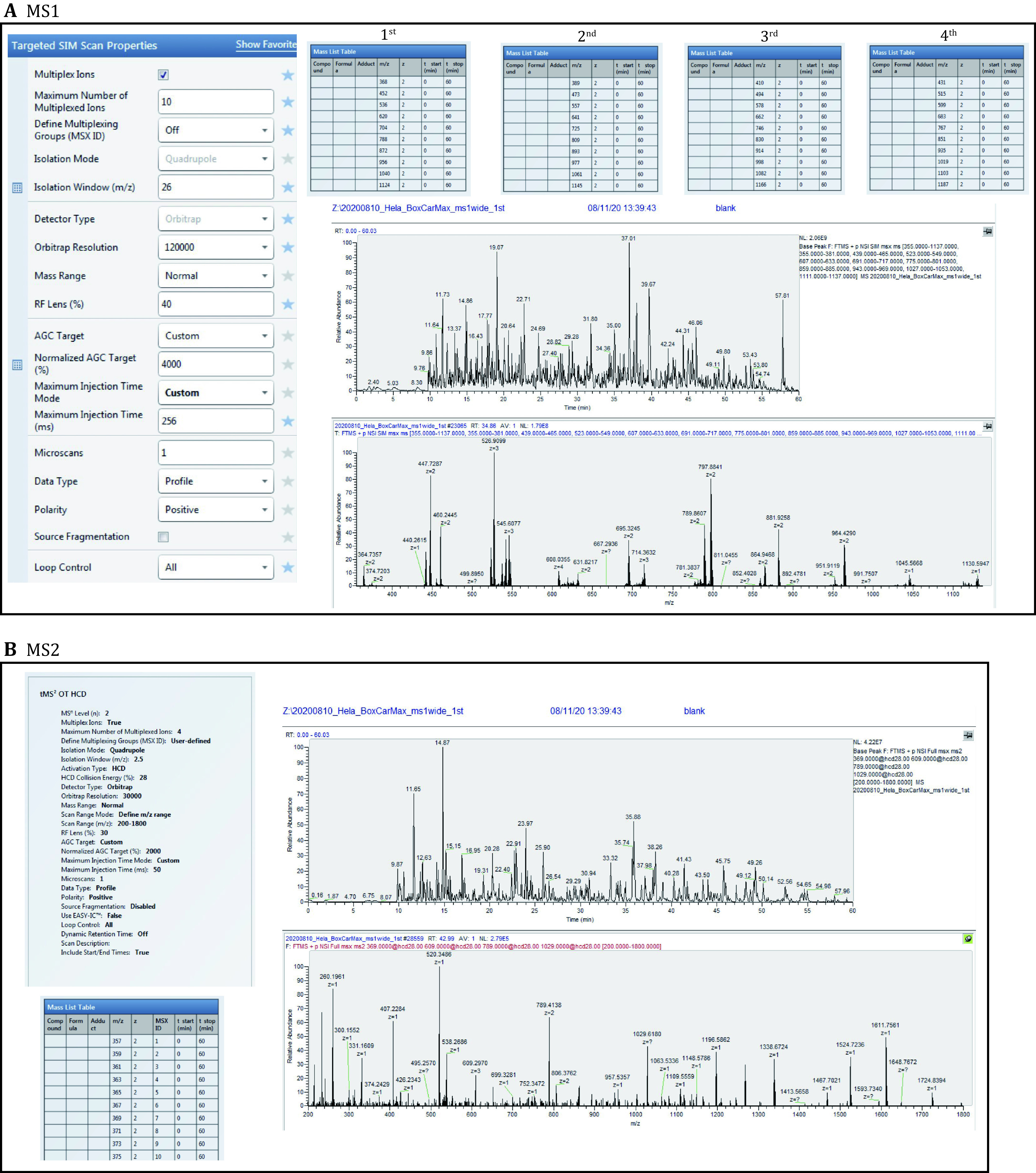
The MS1 and MS2 setting for BoxCarmax-DIA. **A** The MS1 parameters and example of BoxCarmax MS1 (tSIM) scans. Among the four injections for one biological sample, each injection uses 60 min measurement time at a different set of boxcar MS1 windows (26 *m*/*z* isolation width). The representative total ion chromatograph (TIC) cross full MS1 scan and one MS1 spectrum are shown as examples. **B** The MS1 parameters and example of BoxCarmax MS2 scans. The first 10 scanning windows of 2.5 *m*/*z* each, as well as representative base peak chromatograph and one MS2 spectrum for one MSX are shown as examples of MS2 configuration. Please also see supplementary Table S1 for tables directly importable to Xcalibur

6.3 MS1 (tSIM) settings. The MS1 isolation windows is 26 *m*/*z*. One injection: 26 *m*/*z* × 10 windows (see Fig. 3A). Figure 3A also lists the mass acquisition windows (*i*.*e*., the boxcar windows) of the four injections.

**[TIP]** We applied similar rectangle boxcar windows as reported by the Mann lab (Meier *et al*. 2018). However, instead of performing different boxcar window sets in individual MS1 scans, we allocate the different window sets in individual sample injections. According to the original boxcar report, the MS1 sensitivity should be greatly improved, because more ion injection time is used in the mass regions that are less ion intensive (Meier *et al*. 2018).

**[TIP]** Instead of using the 22 *m*/*z* window in the original BoxCarmax-DIA method (Salovska *et al*. 2021), we herein increased the MS1 window size to 26 *m*/*z* to eliminate some software incompatible issues.

6.4 MS2 setting. To configure the MS2 (or MSX) (Egertson *et al*. 2013) scan parameters for BoxCarmax (see Fig. 3B). A total of 120 small windows (each is 2.5 *m*/*z* width) are allocated in 30 MSX scans in one injection. Please use each sheet in the supplementary Table S1 to import the method in Xcalibur.

**[TIP]** MS2 *m*/*z* values are designed to match the MS1 boxcar ranges per injection. In each MSX scan, four small windows are multiplexed, but are kept as far as possible between each other. In this way, all the charge 2+ and 3+ heavy and light SILAC pairs will be separately isolated and measured in different MSX scans, increasing the quantitative accuracy for SILAC labeling peptides (*e*.*g*., b ions can be used) and PTM-carrying peptides.

### Step 6*: A faster version of DIA-MS analysis using a classic DIA-MS method [TIMING ~2 h]

**[TIP]** Alternatively, a regular DIA method of 1.5 or 2 h also can be used for the phosphoproteome data acquisition (Gao *et al*. 2021), especially for low sample amounts of <1–2 μg and large-cohort studies.

6.1* NanoLC setting. Same as the BoxCarmax-DIA method above.

6.2* MS settings. The classic DIA-MS method consists of an MS1 survey scan and 33 MS2 scans of variable windows as described previously (Bruderer *et al*. 2017, 2019). The MS1 scan range is 350–1650 *m*/*z* and the MS1 resolution was 120,000 at *m*/*z* 200. The MS1 full scan AGC target value was set to be 2.0 × 10^6^ and the maximum injection time was 100 ms. The MS2 resolution was set to 30,000 at *m*/*z* 200, and the normalized HCD collision energy was 28%. The MS2 AGC was set to be 1.5 × 10^6^ and the maximum injection time was 50 ms. The default peptide charge state was set to 2. Both MS1 and MS2 spectra were recorded in a profile mode.

### Step 7: Phos-DIA raw data processing in Spectronaut [TIMING ~2 h]

**[TIP]** Below we describe an example in which we use the library-free directDIA pipeline in Spectronaut v17 (Bruderer *et al*. 2017; Tsou *et al*. 2015). Based on our experience, the library-free approach provides comparable results to the library-based analysis.

7.1 Open Spectronaut and “Set up a directDIA Analysis”. Import all LC-MS/MS measurements you want to include in this experiment.

7.2 Select an appropriate protein sequence FASTA database. Note this database must be first imported into Spectronaut using the Database perspective. For most applications, a Swiss-Prot species-specific database is appropriate.

**[TIP]** If not done yet, import the FASTA protein sequence database into Spectronaut prior to starting the analysis. Go to Databases -> Protein Databases -> “Import” in the lower left panel.

7.3 On the left panel, select the “BGS Phospho PTM Workflow” schema. For most phosphoproteomic experiments, use the default settings. However, the following parameters should be double-checked before submitting the analysis.

(A) Pulsar Search -> Peptides. Select a Trypsin/P cleavage.

(B) Pulsar Search -> Modifications. Select cysteine carbamidomethylation as a fixed modification. Set the variable modifications to protein N-terminal acetylation, oxidation of methionine, and S/T/Y phosphorylation.

(C) Pulsar Search -> Identification. PSM, peptide, and protein group false discovery rates (FDR) should be set to 0.01. The directDIA+ (Deep) workflow provides the best identification results.

(D) DIA Analysis -> Quantification. Select precursor filtering based on Qvalue. Cross-Run normalization enabled with automatic normalization strategy (chose the local normalization for samples with number < 500).

(E) DIA Analysis -> Quantification -> Show advanced settings. Interference correction removes ions with high interference scores. It should be enabled with at least two MS1 and three MS2 features retained. Select TOP3 quantification strategy by using the top three fragments for precursor quantification and the top three precursors for peptide quantification.

(F) DIA Analysis -> PTM Workflow. Set to 0.75, this parameter will be modified in post-processing as described below.

7.4 Optional: specify conditions, fractions, and replicates.

7.5 Optional: specify species-specific gene annotation.

7.6 Optional: select other DIA or DDA runs that might improve the proteome coverage depth (such as fractionated samples).

7.7 Double-check all parameters and submit the analysis by clicking on “Finish”.

### Step 8: Export the Spectronaut results [TIMING ~0.5 h]

**[TIP]** We describe an example in which we use the phosphopeptide quantification workflow applying two different localization filters, as described in our previous papers (Gao *et al*. 2021; Salovska *et al*. 2023). We do not use the default PTM Site Report that exports individual P-site quantification but rather export the precursor level report and select the most representative phospho-precursor for each P-site. The phospho-precursor selection is based on the number of missing values and top intensity across all runs. In our hands, this strategy has provided more accurate results.

8.1 Go to reports -> Standard report -> pivot report -> “Peptide Quant” and modify this report by selecting columns as follows.

(A) Row labels: select “PG.ProteinAccessions, PG.Genes, PG.ProteinDescriptions, EG.ProteinPTMLocations, EG.PrecursorId, EG.ModifiedSequence.

(B) Cell Values: select EG.TotalQuantity.

(C) Save Schema for future exports.

8.2 Export Report. This report uses the PTM Localization probability cutoff of 0.75 (*i*.*e*., class I sites; Olsen *et al*. 2006) to export a list of confidently localized precursors -> “PTM075 report”.

8.3 Go to Spectronaut analysis perspective -> Open the experiment settings after double-clicking on the experiment. Go to DIA Analysis -> PTM Workflow and change the PTM Localization Probability Cutoff to 0, then press confirm.

8.4 Export Report using the same export scheme, this report uses the PTM Localization probability cutoff of 0 (*i*.*e*., class I sites; Olsen *et al*. 2006) -> “PTM000 report”. We have made a comparison between two different PTM Localization probability cutoff strategies, and the result indicates that using the PTM probability cutoff of 0.00 improves the data completeness (of the phospho-precursors passing the PTM probability cutoff of 0.75) as well as the sensitivity of statistical analysis (supplementary Fig. S2 and Table S2).

### Step 9: Combine and filter the exported Spectronaut reports

9.1 Filter the “PTM000” report to only contain precursors (EG.PrecursorId) exported in the “PTM075” report. This procedure improves data completeness while only retaining precursors with confident PTM localization with at least one MS run included in the analysis.

9.2 Select only precursors with a phosphorylation site. This information can be found in the EG.ModifiedSequence column.

9.3 Replace relative intensity values lower than 500 by NA, log2 transform the data, and perform additional data normalization, if necessary, based on data quality inspection.

9.4 For each modified sequence (EG.ModifiedSequence) identify the phospho-precursor with the least missing values across all runs. In case there are multiple phospho-precursors with the same number of missing values, select the one with the top intensity based on the average across the runs.

9.5 Use the processed table for downstream data analysis.

**Figure 4:**
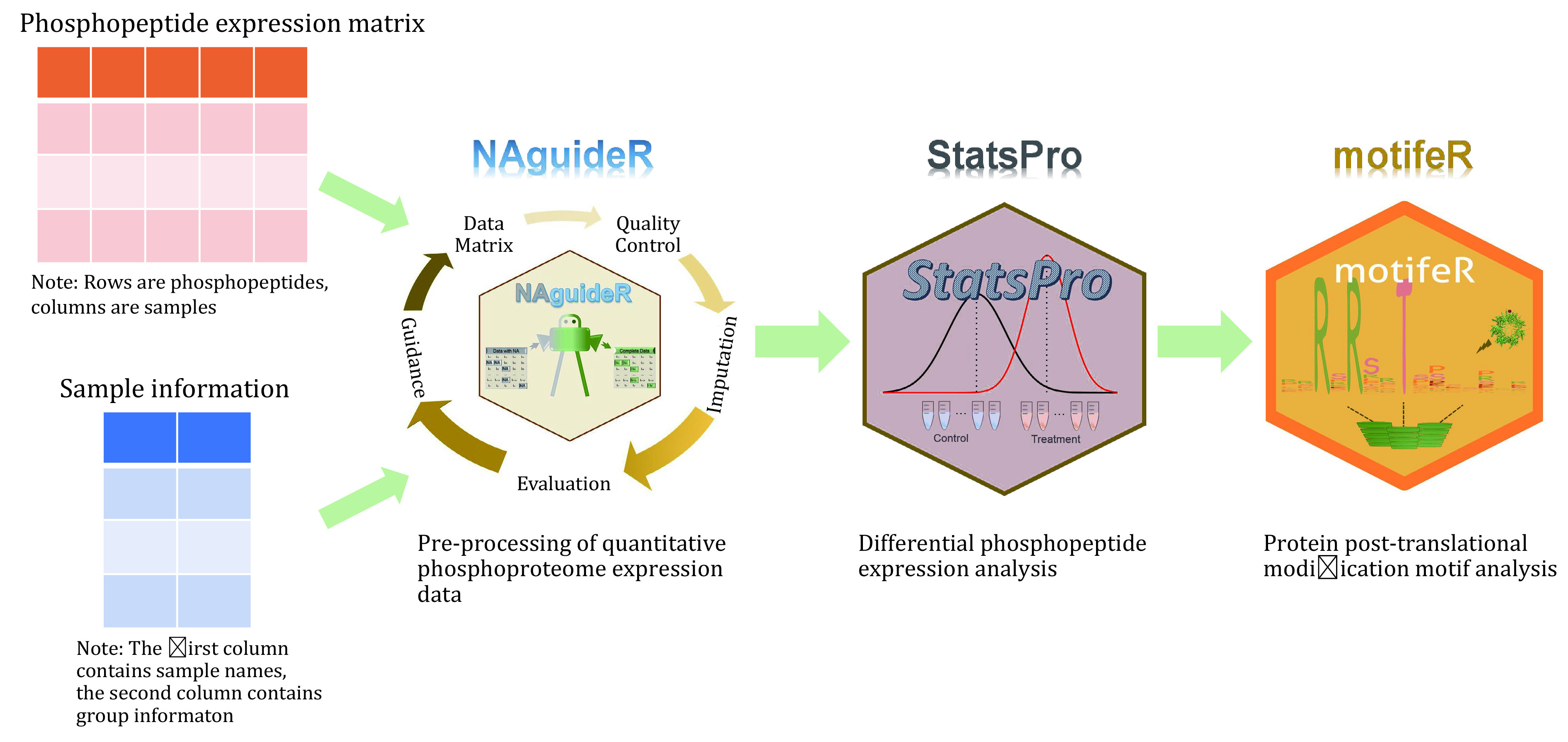
The example of procession and the initial analysis of quantitative phosphoproteome expression data

**[TIP]** The whole basic analysis process of quantitative phosphoproteome expression data can be divided into three steps, which are implemented by three published software (NAguideR, StatsPro and motifeR) (See Steps 11–13, Fig. 4).

### Step 10: P-site-specific enrichment analysis in Perseus [TIMING ~2 h]

10.1 Download P-site-specific information from available databases as described below. We recommend the Omnipath database (Türei *et al*. 2016, 2021), the PhosphoSitePlus database (Hornbeck *et al*. 2019), the PTM signature database (PTMsigDB; Krug *et al*. 2019), or the Signor 2.0 database (Licata *et al*. 2020). For example, download the kinase-substrate association dataset from the PhosphoSitePlus database as follows.

(A) Go to https://www.phosphosite.org/

(B) Downloads -> Datasets from PSP

(C) Download the Kinase_Substrate_Dataset.gz dataset.

(D) Use the SUB_GENE and SUB_MOD_RSD for the mapping of the known kinase-interaction to the phosphorylation dataset.

10.2 Import both the phosphorylation dataset and the annotation datasets to Perseus (Tyanova and Cox 2018; Tyanova *et al*. 2016) and use the row-matching function to combine the tables together using the gene and modified residue ID. Note, the annotation terms need to be uploaded as categorical columns.

10.3 Perform the enrichment analysis using the Fisher’s exact test function in Perseus.

(A) Go to Annot. Columns -> Fisher’s exact test

(B) Select the categorical column that will be used for the grouping, *e*.*g*., containing the information on whether a P-site was up- or down-regulated, and perform the enrichment analysis.

(C) Note: the statistical analysis can be performed both considering unique P-site ids and unique protein ids depending on the analysis. For kinase-substrate enrichment analysis, use the P-site level analysis. To do so, leave the relative enrichment option as <None>.

10.4 For a more sensitive analysis independent of statistical analysis results, the enrichment analysis can be also performed using relative values, such as the log2 fold change between two conditions. In this case, the 1D or 2D enrichment analysis can be performed in Perseus (Cox and Mann 2012).

(A) Go to Columns -> 1D annotation enrichment

(B) Use the columns with the relative values, such as log2 fold change columns.

(C) Note the 2D annotation enrichment can be used to perform the analysis for two different columns, such as log2 fold change based on different comparisons or measured at different molecular layers.

### Step 11: Pre-processing of quantitative phosphoproteome expression data in NAguideR (https://github.com/wangshisheng/NAguideR) [TIMING ~2 h]

11.1 Software startup. Users can visit this site directly: http://www.omicsolution.com/wukong/NAguideR. Optionally, users can also run this tool on their computer and install it as described on the GitHub: https://github.com/wangshisheng/NAguideR.

11.2 Data preparation. Prepare two required data: (1) Phosphopeptide expression table. There are currently four types of proteomics expression data supported in NAguideR (*i*.*e*., 'Peptides + Charges + Proteins', 'Peptides + Charges', 'Peptides + Proteins', 'Proteins'), among which the main differences are the first few columns.

**[TIP]** In addition, users may upload other kinds of omics data (*e*.*g*., genomics, metabolomics), for which they can just need to choose the fifth type ('Others'); (2) Sample information, which means that users should provide sample group identity information. This information could, *e*.*g*., enable filtration strategies for different groups respectively in a later step (see below). The sample names are in the first column and their orders are the same as those in the expression data. Group information is in the second column.

11.3 Missing value filtering. Firstly, users should understand what the missing values look like in the expression data. This software will recognize these missing values and replace them with NAs. Then they can check the missing value situation of their data and filter those data with a high proportion of missing values in this step. Usually, the proportion of missing values is set to 50%, which means those phosphopeptides with more than half of the missing values among all samples will be removed.

11.4 Data normalization. Users can normalize their data in this step. By default, NAguideR will process median normalization for original data.

11.5 Coefficient of variation (CV). Those phosphopeptides with CV above some threshold will be removed. The threshold is usually set to 0.3.

11.6 Logarithm transformation. The data will be transformed to the logarithmic scale with base 2.

11.7 Missing value imputation. Select any of the 23 missing value imputation methods supported in NAguideR.

**[TIP]** All methods are classified into three categories based on their algorithm (Single value approaches, global structure approaches and local similarity approaches). After selecting suitable methods, users can process missing value imputation.

11.8 Results and assessments. Obtain the imputed results and choose the best one based on the four classic criteria (NRMSE, SOR, ACC_OI, PSS) and/or the four proteomic criteria (Charge, PepProt, CORUM and PPI).

### Step 12: Additional differential phosphopeptide expression analysis in StatsPro (optional) (https://github.com/YanglabWCH/StatsPro) [TIMING ~1 h]

12.1 Software startup. Users can click this site directly: http://www.omicsolution.com/wukong/StatsPro.

12.2 Data Preparation. Here users can use the results from Step 11 (the imputed expression data and the sample information). In the expression data, protein ids/names and peptides number are sequentially provided in the first two columns.

12.3 Data Pre-processing, which means Missing value filtration, Data normalization, CV, and Missing value imputation. As users have finished these steps in Step 10, they can ignore these steps here.

12.4 Statistical analysis. Choose any of 12 common statistical methods (I. Parametric tests, including *t-*test, one-way ANOVA, limma and SAM. II. Non-parametric tests, including Wilcoxon rank sum test, Kruskal-Wallis rank sum test, permutation test, RP, ROTS, MSqRobSum, DEqMS and PLGEM) in this software.

12.5 Results and assessments. Obtain the proper statistical results based on the three criteria (Number of detections, the correlation coefficient between *P*-values and effect sizes, AUC and F1 score) and filter the results.

### Step 13: Protein post-translational modification motif analysis in motifeR (https://github.com/wangshisheng/motifeR) [TIMING ~1 h]

13.1 Software startup. Users can open this site in their browser directly: http://www.omicsolution.com/wukong/motifeR. Or, users can install this tool on their computer as described on the GitHub: https://github.com/wangshisheng/motifeR.

13.2 Data Preparation. Prepare two required data: (1) Peptide sequences with post-translational modification (*e*.*g*. phosphorylation). Users can use the differentially expressed phosphopeptide sequences from Step 12, or the whole phosphopeptide sequences without statistical analysis from Step 11. Please note the modified residues should be marked with some label, such as “#” or “@”. (2) Background database contains the whole protein sequences which can be downloaded from UniProt (http://www.uniprot.org/) as a FASTA sequence database.

13.3 Set some basic parameters, for example, Central amino acid (the central residue that users want to analyze. For phosphorylation motif analysis, it can be phosphorylated S, T or Y residue); Label of modification: the label represents modification, users can use some label they like, such as “#”, “@”, where “#” is recommended; Width (the number of left/right side characters of the central residue, the default value is 7). Minimum number: this threshold refers to the minimum number of peptides (default is *n* = 20) you wish in each of your extracted motifs to occur in the data set. *P*-value threshold: the *P*-value threshold for the binomial probability. This is used for the selection of significant residue/position in the motif.

13.4 Pre-alignment. Align those peptide sequences with the background database (protein sequences) and force the modified sites/residues to be central sites. Therefore, users can get the standard peptide window sequences, whose lengths are calculated according to the parameter width in Step 13.2. In addition, this software can count the number of modified sites and plot the distribution. Then this tool also extracts those peptides with multi-modified sites so that users can choose to perform additional analysis according to this result in the next step.

13.5 Motif enrichment. Find the overrepresented sequence motifs among the phosphopeptide sequences. It's worth noting that if users choose the “Only use multi-site data” parameter, this tool will only take the peptides with multi-modification sites as foreground data, that is, it will use the sequences in the Seqwindows_MultiSites column obtained from “Pre-alignment” step as foreground data.

13.6 Motif plot. Plot the motifs from the enrichment results. Users can type in one number or two numbers connected by a horizontal line (*e*.*g*. 1–10) in the “Motif index for plot” parameter, it will plot the relative motif. In addition, if users select the “Equal height or not?” parameter, all residues in the plot will have equal height.

13.7 Kinase-substrate analysis. Infer relative kinase activities based on the PhosphoSitePlus and NetworKIN databases.

**[TIP]** Users should note here: (1) There are only human databases for this analysis in this system currently; (2) This Kinase-substrate analysis is only for phosphoproteomics data, other modification data are not appropriate. Finally, this software gives a result table containing kinases and substrates information and plots the kinase-substrate network.

## CONCLUDING REMARKS

**Figure 5:**
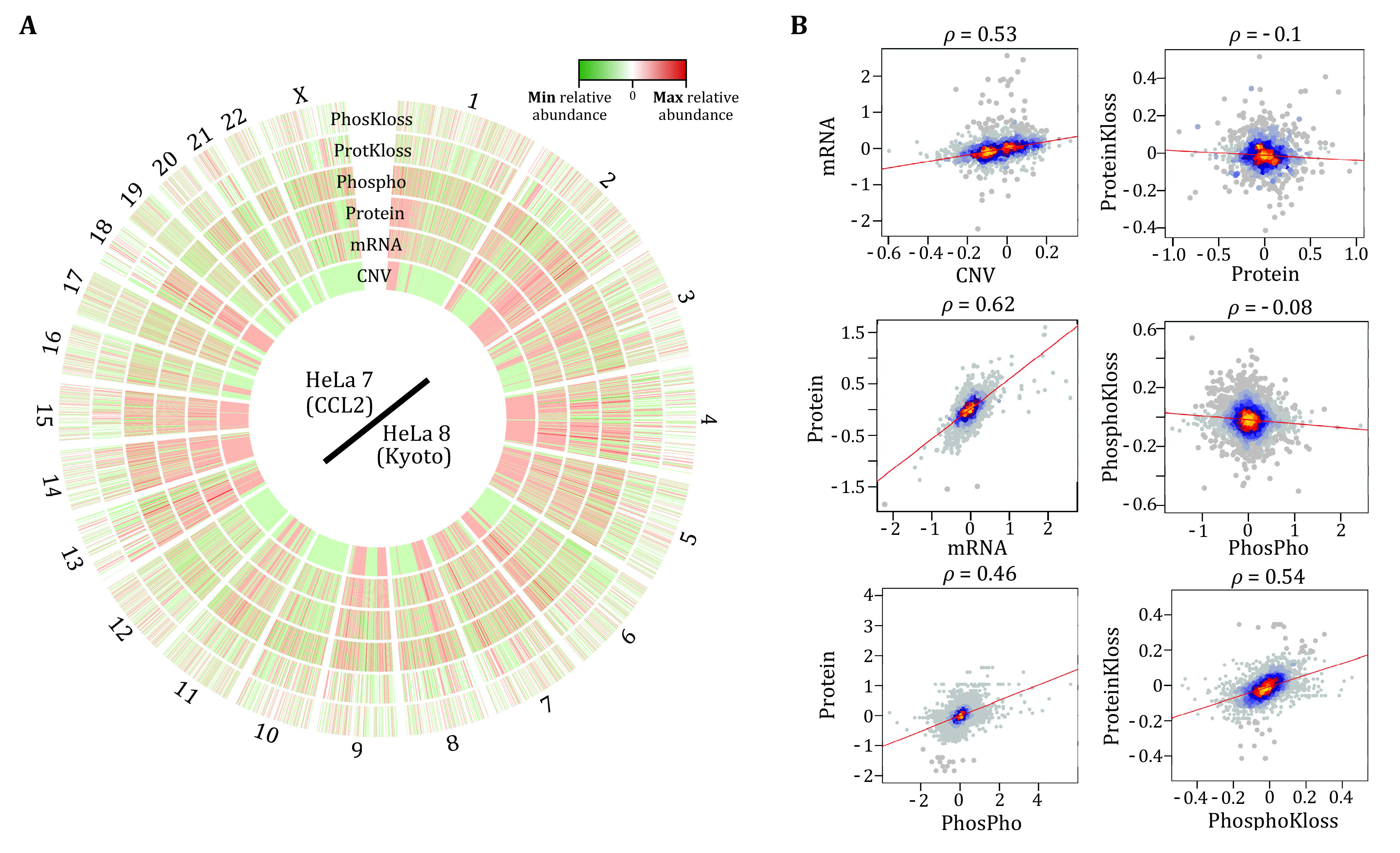
Phosphoproteome-centric relative-scale correlation analysis between different molecular layers provides valuable insights into mechanisms determining cellular functions. **A** Circos plot of HeLa 7 (CCL2)/HeLa 8 (Kyoto) ratios at CNV, mRNA, protein and phosphorylation abundance, and protein and phosphorylation turnover rates (*i*.*e*. Kloss). Fold changes from high to low are shown in red to green. The data are phosphoproteome centric, *i*.*e*., data matched to available phosphoproteomic identifications. **B** Spearman correlation analysis between different layers using HeLa7/HeLa8 ratio data. Spearman’s rho is shown

In this Protocol contribution, we describe an optimized Phosphoproteomic-DIA workflow that enables precise quantification of thousands of phosphosites based on our recent experience, studies, and developed methods. Like with any data generation efforts for MS-based proteomics, we found that sample quality (*e*.*g*., phosphopeptide enrichment efficiency), liquid chromatography conditions (*e*.*g*., analytical column efficiency for DIA-MS), and stable MS running status are crucial factors for satisfactory phosphoproteome coverage. For example, sharp chromatographic peaks can increase the phosphopeptide resolution on the retention time dimension and improve identification rates, similar to the scenario of analyzing non-PTM peptides. However, the successful phosphoproteomic analysis never lies in expensive MS instruments, not even in the analytical depth of the entire phosphoproteome, but in the biological insights and evidence acquired that support new discoveries in systems biology and systems medicine. In fact, our described protocol ends at the initial NA imputation, motif extraction, and differential phosphosite extraction. Practically, the further phosphoproteomic data analysis tailored to particular projects can be much more challenging and exciting after these initial steps. This step is much less mature and cannot be described as a general and simple protocol. For example, using Phos-DIA and DeltaSILAC, we have measured the steady-state phosphoproteomes of two HeLa cell strains (CCL2 vs. Kyoto) with 25,000 phosphosites (Wu *et al*. 2021). The further Circos analysis of this dataset enables a phosphoproteome-centric relative-scale correlation analysis between different molecular layers, underscoring both functional heterogeneities between the two HeLa stains and the underlying determining factors of such heterogeneity via gene expression and post-translational regulation (Fig. 5). Moreover, a certain longitudinal phosphoproteomic dataset might benefit from *e*.*g*., fuzzy c-clustering analysis (Conesa *et al*. 2006) and other signaling reconstruction algorithms (Gjerga *et al*. 2021; Kim *et al*. 2021). Due to the current sparse understanding of all phosphosites discovered by MS, the PTM site-specific annotation databases (Krug *et al*. 2019) and bioinformatic framework incorporating new phosphoproteomic results will be highly appreciated in the future (Liu 2022; Ochoa *et al*. 2020; Xiao *et al*. 2022; Yang *et al*. 2015). Finally, we hope this Protocol contribution could be helpful, particularly for new phosphoproteomic users in designing and implementing their experiments.

## Conflict of interest

Yi Di, Wenxue Li, Barbora Salovska, Qian Ba, Zhenyi Hu, Shisheng Wang and Yansheng Liu declare that they have no conflict of interest.
